# The causal relationship between white blood cell counts and hepatocellular carcinoma: a Mendelian randomization study

**DOI:** 10.1186/s40001-022-00900-y

**Published:** 2022-12-06

**Authors:** Guo-Qiang Pan, Chun-Cheng Yang, Xiao-ling Shang, Zhao-Ru Dong, Tao Li

**Affiliations:** 1 Department of General Surgery, Qilu Hospital, Shandong University, China Jinan,; 2 Department of Medical Oncology, Qilu Hospital, Shandong University, Jinan, China; 3 Department of Hepatobiliary Surgery, The Second Hospital of Shandong University, Jinan, China

**Keywords:** HCC, White blood cells, Mendelian randomization study, Causal relationship

## Abstract

**Background:**

Most of hepatocellular carcinoma (HCC) arises on the background of chronic inflammation. The presence of infiltrating inflammatory cells is associated with tumour initiation, progression and clinical response to treatment. The influence of white blood cell (WBC) subtype counts on HCC progression remains unclear.

**Methods:**

In this study, we performed a Mendelian randomization (MR) study with the validation of two datasets. The summary data for WBC counts were extracted from a recent large GWAS of individuals of European ancestry. The GWAS data related to HCC were obtained from the UK Biobank (UKB). Univariable and multivariable MR analyses were used to identify risk factors genetically associated with HCC risks.

**Results:**

In the discovery dataset, multivariable MR analysis revealed that sum basophil neutrophil counts had an independent causal effect on the occurrence of HCC, with the sum basophil neutrophil counts as follows: (OR = 0.437, *P* = 0.003, CI 0.252–0.757). Similarly, in the validation dataset, total basophil neutrophil counts were also been identified as an independent risk factor for HCC. The sum basophil neutrophil counts were as follows: (OR = 0.574, *P* = 0.021, CI 0.358–0.920).

**Conclusion:**

In the European population, genetically predicted lower total basophil neutrophil counts might be an independent risk factor for HCC.

## Introduction

Liver cancer is the fourth leading cause of cancer-related deaths worldwide [1]. Hepatocellular carcinoma (HCC), which accounts for more than 80% of primary liver cancer, is an aggressive tumour that frequently occurs in the setting of cirrhosis and chronic liver disease [2, 3]. For HCC patients, surgical resection is one of the most effective treatments. Despite improvements in treatment strategies, the 5-year survival rate of HCC patients remains unsatisfying compared with that of other cancers patients [4].

Various inflammatory factors play an important role in tumour growth, progression, angiogenesis, and metastases. Notably, white blood cell (WBC) counts are widely accepted biomarkers of systematic inflammation. WBCs mainly comprised five subtypes, including neutrophils, lymphocytes, monocytes, basophils and eosinophils. Inflammatory markers derived from blood samples, such as the neutrophil-to-lymphocyte ratio (NLR), lymphocyte-to-monocyte ratio (LMR) and platelet-to-lymphocyte ratio (PLR), have been identified as potentially valuable prognostic markers in patients with various types of cancer, including HCC [5–8]. For example, pretreatment peripheral neutrophils, lymphocytes, and monocytes are reported to be independently associated with the outcomes of HCC patients [9]. Growing evidence shows that neutrophils play an important role in the HCC pathogenesis such as tumorigenesis, local tumour progression and metastasis [10]. Several clinical studies demonstrated that elevated neutrophil count and increased NLR are markers of advanced disease, poor prognosis and poor response to therapy in HCC [11–14]. In regard to causal inference, observational studies may be biased by confounding factors and reverse causality. Since the causal associations between WBC subtypes and HCC risk have not been thoroughly investigated, identifying host factors predisposing individuals to HCC is urgently needed to improve primary prevention and develop treatment strategies.

Mendelian randomization (MR) studies, which use genes as instrumental variables (IVs) to research disease associations, can effectively solve the confounding and reverse causation associated with traditional observational studies [15, 16]. Because genetic variations are randomly inherited from parents to offspring during pregnancy, these genetic variations are unlikely to be affected by potential confounding factors and reverse causality. Genome-wide association studies (GWAS) have identified hundreds of single nucleotide polymorphisms (SNPs) associated with HCC-related traits and WBC subtypes [17–19], which creates an opportunity to use the MR approach to test genetic and potential causal relationships between WBC subtype count and HCC risk.

Our present study attempted to use MR analysis to identify SNPs strongly related to blood WBC subtype counts to evaluate the causal association between WBC subtypes and HCC risk.

## Methods

### Summarized statistics of WBC counts from a genome-wide association study (GWAS)

The GWAS summary statistics of the WBC counts in our study included 8 phenotypes: eosinophil counts, basophil counts, neutrophil counts, lymphocyte counts, monocyte counts, sum eosinophil basophil counts, sum neutrophil eosinophil counts and sum basophil neutrophil counts. The WBC count-related SNPs were obtained from a recent large GWAS of European ancestry patients. The sum of white blood cell counts was defined as follows: first, the corresponding white blood cell counts were added, and then the data were transformed into SD [18]. The large GWAS included a total of 173,480 European ancestry individuals from three large-scale UK studies—INTERVAL (*n* = 40,521), approved by the Cambridge (East) Research Ethics Committee, UK Biobank (*n* = 87,265) and UK BiLEVE (a selected subset of the UK Biobank cohort, *n* = 45,694). These GWASs tested univariate associations of 36 indices with 29.5 million imputed variants that passed quality control filters (MAF > 0.01%) and used stepwise multiple regression to identify a parsimonious subset of genetic variants explaining the genome-wide significant associations for each trait. Our study expands the repertoire of genes and regulatory mechanisms governing haematopoietic development in humans and opens potential avenues for targeting key pathways involved in abnormal or dysregulated haematopoiesis (Table 1).

**Table 1:** Summary of white cell counts

Exposure	NSNP	Unit	Sample	R^2^	F	PMID
Eosinophil counts	179	SD	172,275	3.48	83.65	27863252
Basophil counts	81	SD	171,846	1.24	68.63	27863252
Neutrophil counts	154	SD	170,702	12.33	74.67	27863252
Lymphocyte counts	171	SD	171,643	6.72	76.93	27863252
Monocyte counts	201	SD	170,721	33.86	115.78	27863252
Sum eosinophil basophil counts	175	SD	171,771	2.76	81.27	27863252
Sum neutrophil eosinophil counts	151	SD	170,384	9.05	75.67	27863252
Sum basophil neutrophil counts	181	SD	170,143	5.57	106.39	27863252

SD is the standard deviation

### Extraction of SNPs associated with HCC

We extracted the 2 summary GWAS statistics of HCC from the UK Biobank, which included 456,348 and 456,276 individuals of European ancestry, and the study adjusted for age, age squared, and study-specific covariates (UKB. The disease codes for hepatocellular carcinoma in the UK Biobank are “ICD 10, C22.0” and “Data-Field 20001_1024”) [20]. When assessing the causality between exposures and HCC, the summary statistics of HCC with “ICD 10, C22.0” were initially used as the discovery set, and the “Data-Field 20001_1024” GWAS was used for validation. In univariable MR analysis, we simply estimated the relationship between each risk factor and HCC. In the multivariable MR analysis, we tried to identify the independent risk factors of HCC.

### Mendelian randomization design and instrumental variables selection

**Fig. 1:**
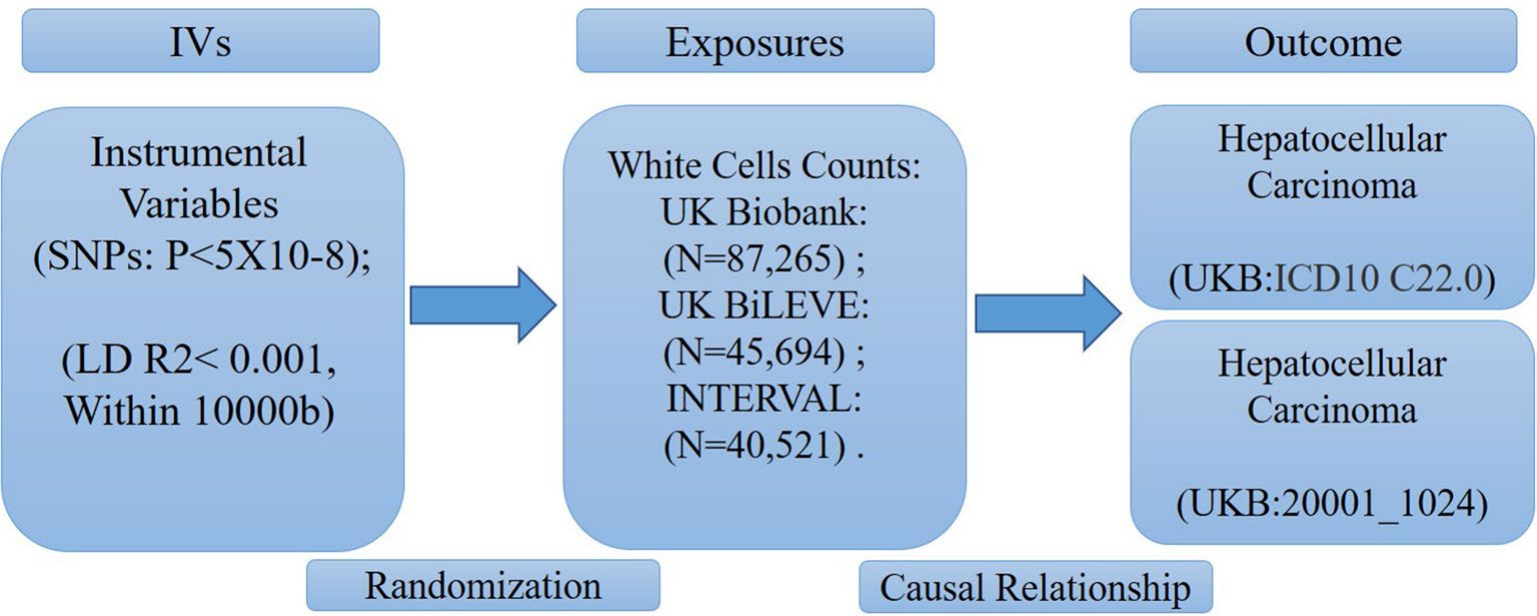
The main design of this MR study

In MR, genetic variant(s) are used as IVs for assessing the causal effect of the exposure on the outcome. The fundamental conditions for a genetic variant to satisfy an IV are summarized as follows: (1) the variant is associated with the exposure; (2) the variant is not associated with any confounder of the exposure–outcome association; (3) the variant does not affect the outcome, except possibly via its association with the exposure. We selected the significant genetic variants associated with the exposures of interest from the GWAS (significance level *p* < 5 × 10^−8^). The minor allele frequency of the SNP was  > 0.01. Then, the SNPs used in our study satisfied linkage disequilibrium (LD, r^2^ < 0.001, kb > 10,000) in the given genome region, and the SNPs with palindromic structures were removed. F statistics (F = beta^2^/se^2^) were used to evaluate the remaining SNP power, so we calculated F statistics for each SNP. The SNPs with F statistics  < 10 were identified as weak instruments, and then we excluded them (Fig. 1).

### Mendelian randomization analysis and sensitivity test

For the univariable MR, the inverse variance weighted (IVW) method, the MR-Egger method and the weighted median (WM) were used to estimate the effect of the exposures on outcome. For multivariable MR, we used regression-based IVW, a method of weighted averaging of random variables. In this study, IVW was the main method adopted in the statistical analysis. MR-Egger and weighted-median (WM) methods were used as supplements to the IVW method.

We performed the MR-PRESSO global test, outlier test, and distortion test to identify and remove SNPs with horizontal pleiotropy. If any outliers existed, we repeated the evaluation of causal relationships. The intercept test of MR-Egger and Cochran’s *Q* test in IVW and the MR-Egger model were used to assess pleiotropy and heterogeneity [16]. In the case of pleiotropy, we prefer to use the MR-Egger. If the *P* value in Cochran’s *Q* test was significant (*P* < 0.05), the WM model was used to analyse the statistics. Otherwise, a fixed-effects model was used. Furthermore, we conducted a leave-one-out analysis. The statistical power were tested by online approval (https://cnsgenomics.shinyapps.io/mRnd/).

Genetic variants associated with exposures at genome-wide significance (*P* < 5 × 10^−8^) were then LD-pruned (distance threshold = 10,000 kb, r^2^ = 0.001) using the clump_data command in the “TwoSampleMR” package in R to identify an independent set of variants to serve as a genetic instrument for exposures. Univariable MR analysis was performed by the R packages “Two Sample MR” and “Mendelian randomization”. Multivariable MR was performed by the R packages “MVMR” and “Mendelian randomization”. MR-PRESSO was conducted using the R package “MRPRESSO”. Data visualization was conducted using R software 4.1.1 (https://www.r-project.org/).

## Result

Among the exposures of WBC counts, the genetically predicted counts of most WBCs may not be associated with the risk of HCC except the sum basophil neutrophil counts, based on both datasets. Lower total basophil neutrophil counts may be an independent risk factor for HCC. Univariable MR analysis based on one dataset suggested that eosinophil counts had a significant association with HCC, but the association became nonsignificant after adjustments for other traits.

### Univariable MR analysis of exposures on HCC risks in the discovery stage

To characterize the relationship between WBC counts and the risk of HCC, we constructed a genetic instrument for WBC counts using 73–173 independent SNPs associated with the above 8 traits at a genome-wide level of significance (*P* < 5 × 10^−8^), which accounted for 1.24–33.86% of the variability in the WBC counts. The mean F-statistic ranged from 68.63 to 115.78, suggesting that the risk of weak instrument bias was low. Univariable MR analysis identified a lower sum of basophil neutrophil counts as a risk factor for HCC. Briefly, each 1-SD increase in the sum basophil neutrophil count could help reduce the risk of HCC (OR = 0.400, *P* = 0.0001, CI 0.233–0.684). We found no strong evidence to support causal associations between HCC risk and the other WBC subtypes including eosinophil counts (OR = 0.560, *P* = 0.059, CI 0.307–1.022), basophil counts (OR = 1.039, *P* = 0.939, CI 0.384–2.811), neutrophil counts (OR = 1.144, *P* = 0.832, CI 0.328–3.989), lymphocyte counts (OR = 0.636, *P* = 0.198, CI 0.320–1.266), monocyte counts (OR = 1.213, *P* = 0.472, CI 0.717–2.051), sum eosinophil basophil counts (OR = 0.691, *P* = 0.236, CI 0.375–1.273), and sum neutrophil eosinophil counts (OR = 0.806, *P* = 0.544, CI 0.403–1.614) (Table 2).

**Table 2:** The effect estimates, test of heterogeneity and test o﻿f pleiotropy of white cell counts on HCC (discovery)

Exposure	NSNP	MR methodology	Effect Estimates HCC			*P* value	Test of heterogeneity		Test of pleiotropy	
			OR	95% LCI	95% UCI		Cochrane *Q* test	Pheterogeneity	MR-Egger intercept	Ppleiotropy
Eosinophil counts	163	IVW	0.560	0.307	1.022	0.059	149.961	0.742		
		MR Egger	0.559	0.136	2.295	0.421	149.961	0.723	< 0.001	0.997
		Weighted median	0.492	0.191	1.268	0.142				
Basophil counts	73	IVW	1.039	0.384	2.811	0.939	76.405	0.339		
		MR Egger	1.098	0.126	9.598	0.933	76.401	0.309	− 0.002	0.955
		Weighted median	1.055	0.224	4.976	0.946				
Neutrophil counts	135	IVW	0.737	0.368	1.476	0.389	165.929	0.032		
		MR Egger	1.589	0.285	8.875	0.406	164.740	0.032	− 0.031	0.329
		Weighted median	1.144	0.328	3.989	0.832				
Lymphocyte counts	145	IVW	0.636	0.320	1.266	0.198	154.957	0.251		
		MR Egger	0.301	0.043	2.101	0.228	154.245	0.246	0.029	0.418
		Weighted median	0.666	0.212	2.099	0.488				
Monocyte counts	173	IVW	1.213	0.717	2.051	0.472	167.025	0.593		
		MR Egger	0.844	0.315	2.260	0.736	166.300	0.587	0.019	0.395
		Weighted median	1.170	0.512	2.673	0.709				
Sum eosinophil basophil counts	162	IVW	0.691	0.375	1.273	0.236	167.036	0.574		
		MR Egger	0.765	0.186	3.145	0.711	157.012	0.552	− 0.004	0.877
		Weighted median	0.549	0.207	1.455	0.228				
Sum neutrophil eosinophil counts	134	IVW	0.806	0.403	1.614	0.544	160.745	0.051		
		MR Egger	1.300	0.233	7.246	0.765	160.297	0.047	− 0.019	0.544
		Weighted median	1.155	0.331	4.030	0.821				
Sum basophil neutrophil counts	157	IVW	0.400	0.233	0.684	< 0.001	174.065	0.143		
		MR Egger	0.342	0.113	1.034	0.059	174.750	0.132	0.008	0.749
		Weighted median	0.450	0.188	1.078	0.445				

NSNP is the number of single nucleotide polymorphism; Odds ratio (OR) is per 1-SD increase; 95% LCI is the lower limit of 95% confidence interval; 95% UCI is the upper limit of 95% confidence interval; *p* value is the *p*-value of OR; Pheterogeneity is the *p*-value of Cochrane’s *Q* value in heterogeneity test; Ppleiotropy is the *p*-value of MR-Egger intercept. IVW is IVW with a fixed-effects model

### Multivariable MR analysis of exposures on HCC risks in the discovery stage

**Fig. 2:**
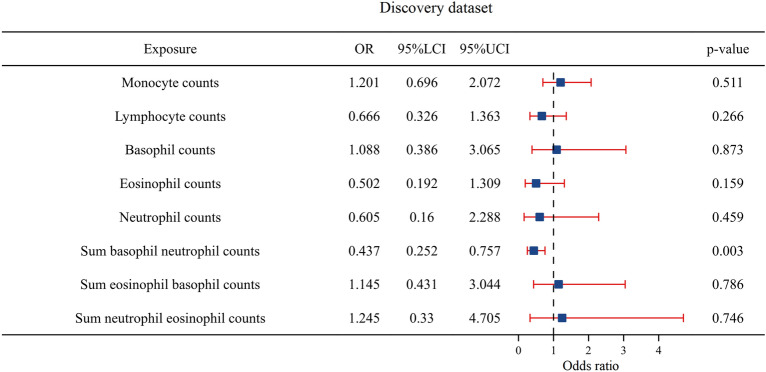
The forest plot of the multivariable Mendelian randomization results based on the discovery dataset. Odds ratio (OR) is per 1-SD increase; 95% LCI is the lower limit of the 95% confidence interval; 95% UCI is the upper limit of the 95% confidence interval

Then, we explored the causal relationship between WBC counts and HCC by conducting a multivariable MR analysis. We observed that sum basophil neutrophil counts had an independent causal effect on the occurrence of HCC: eosinophil counts (OR = 0.502, *P* = 0.159, CI 0.192–1.309), basophil counts (OR = 1.088, *P* = 0.873, CI 0.386–3.065), neutrophil counts (OR = 0.605, *P* = 0.459, CI 0.160–2.288), lymphocyte counts (OR = 0.666, *P* = 0.266, CI 0.326–1.363), monocyte counts (OR = 1.201, *P* = 0.511, CI 0.696–2.072), sum eosinophil basophil counts (OR = 1.145, *P* = 0.786, CI 0.431–3.044), and sum neutrophil eosinophil counts (OR = 1.245, *P* = 0.746, CI 0.330–4.705). The OR of HCC decreased per 1-SD increase in the sum basophil neutrophil counts (OR = 0.437, *P* = 0.003, CI 0.252–0.757) (Fig. 2).

### Univariable MR analysis of exposures on HCC risks in validation stage

In the validation stage, we successfully replicated the MR results of WBC counts. We validated these risk factors for HCC by MR analysis. Univariable MR results demonstrated that sum basophil neutrophil counts had a significant causal effect on HCC. The OR of HCC decreased per 1-SD increase in the sum basophil neutrophil counts (OR = 0.573, *P* = 0.018, CI 0.361–0.909). Eosinophil counts had a weak protective effect on HCC: eosinophil counts (OR = 0.568, *P* = 0.033, CI 0.338–0.954). Other WBC subtypes have no causal relationship with HCC risk: basophil counts (OR = 0.509, *P* = 0.123, CI 0.215–1.202), neutrophil counts (OR = 0.817, *P* = 0.509, CI 0.449–1.488), lymphocyte counts (OR = 0.944, *P* = 0.851, CI 0.521–1.711), monocyte counts (OR = 0.671, *P* = 0.085, CI 0.426–1.056), sum eosinophil basophil counts (OR = 0.596, *P* = 0.054, CI 0.352–1.009), and sum neutrophil eosinophil counts (OR = 0.693, *P* = 0.230, CI 0.381–1.262) (Table 3).

**Table 3:** The effect estimates, test of heterogeneity and test of pleiotropy of white cell counts on HCC (validation)

Exposure	NSNP	MR methodology	Effect Estimates HCC				Test of Heterogeneity		Test of Pleiotropy	
			OR	95% LCI	95% UCI	*P* value	Cochrane *Q* test	Pheterogeneity	MR-Egger intercept	Ppleiotropy
Eosinophil counts	163	IVW	0.568	0.338	0.954	0.033	185.025	0.104		
		MR Egger	0.196	0.054	0.715	0.015	181.455	0.129	0.047	0.077
		Weighted median	0.454	0.202	1.022	0.057				
Basophil counts	73	IVW	0.509	0.215	1.202	0.123	74.866	0.385		
		MR Egger	0.151	0.024	0.938	0.046	72.617	0.424	0.047	0.143
		Weighted median	0.677	0.181	2.537	0.563				
Neutrophil counts	135	IVW	0.817	0.449	1.488	0.509	137.905	0.391		
		MR Egger	0.561	0.144	2.186	0.406	137.525	0.376	0.015	0.545
		Weighted median	0.527	0.183	1.522	0.237				
Lymphocyte counts	145	IVW	0.944	0.521	1.711	0.851	142.808	0.512		
		MR Egger	1.155	0.230	5.809	0.861	142.739	0.490	− 0.008	0.793
		Weighted median	1.075	0.428	2.704	0.878				
Monocyte counts	173	IVW	0.671	0.426	1.056	0.085	174.197	0.439		
		MR Egger	0.418	0.178	0.982	0.047	172.535	0.453	0.025	0.201
		Weighted median	0.517	0.238	1.121	0.095				
Sum eosinophil basophil counts	162	IVW	0.596	0.352	1.009	0.054	186.616	0.082		
		MR Egger	0.156	0.042	0.571	0.006	180.917	0.123	0.002	0.936
		Weighted median	0.380	0.163	0.885	0.025				
Sum neutrophil eosinophil counts	134	IVW	0.693	0.381	1.262	0.230	143.089	0.260		
		MR Egger	0.658	0.162	2.683	0.561	143.082	0.241	0.002	0.936
		Weighted median	0.523	0.183	1.494	0.226				
Sum basophil neutrophil counts	157	IVW	0.573	0.361	0.909	0.018	147.495	0.632		
		MR Egger	0.284	0.117	0.690	0.006	146.179	0.682	0.037	0.071
		Weighted median	0.331	0.158	0.695	0.004				

NSNP is the number of single nucleotide polymorphism; Odds ratio (OR) is per 1-SD increase; 95% LCI is the lower limit of 95% confidence interval; 95% UCI is the upper limit of 95% confidence interval; *P* value is the *P*-value of OR; Pheterogeneity is the *P*-value of Cochrane’s *Q* value in heterogeneity test; Ppleiotropy is the *P*-value of MR-Egger intercept. IVW is IVW with a fixed-effects model

### Multivariable MR analysis of exposures on HCC risks in validation stage

**Fig. 3:**
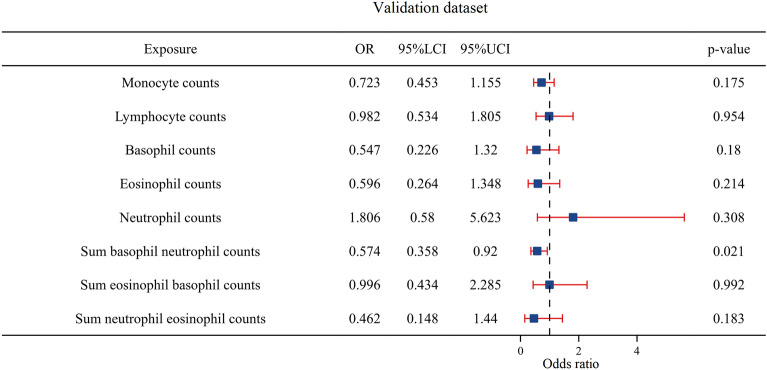
The forest plot of the multivariable Mendelian randomization results based on the validation dataset. Odds ratio (OR) is per 1-SD increase; 95% LCI is the lower limit of the 95% confidence interval; 95% UCI is the upper limit of the 95% confidence interval

Multivariable MR analysis in validation stage also revealed that the sum of the basophil neutrophil counts was an independent HCC risk factor. It is worth noting that after adjustments for other traits, the association between eosinophil counts and HCC became nonsignificant: eosinophil counts (OR = 0.596, *P* = 0.214, CI 0.264–1.348), basophil counts (OR = 0.547, *P* = 0.180, CI 0.226–1.320), neutrophil counts (OR = 1.806, *P* = 0.308, CI 0.580–5.623), lymphocyte counts (OR = 0.982, *P* = 0.954, CI 0.535–1.805), monocyte counts (OR = 0.723, *P* = 0.175, CI 0.453–1.155), sum eosinophil basophil counts (OR = 0.996, *P* = 0.992, CI 0.434–2.285), sum neutrophil eosinophil counts (OR = 0.462, *P* = 0.183, CI 0.148–1.440), and sum basophil neutrophil counts (OR = 0.574, *P* = 0.021, CI 0.358–0.920) (Fig. 3).

### Sensitivity analysis

We observed that the confidence interval of the exposures was relatively wide, which was considered to be caused by low sample size. It cannot be ruled out that there would be weak connections between the other WBC count traits and HCC. Another possibility of the null findings observed in our MR analyses could be explained by the low proportion of variances in some of the exposures (F statistics  < 100). On the other hand, we deemed that sum basophil neutrophil counts had a causal relationship with HCC.

There was heterogeneity of neutrophil counts (*P* < 0.05) in the discovery stage. All the results of these risk factors were the MR-PRESSO corrected results if outliers were detected. No significant horizontal pleiotropic effects were detected in the MR-Egger test (for the intercept of MR-Egger, all *P* values were more than 0.05). The statistical power of these exposures was 100%.

## Discussion

To our knowledge, this is the first MR study evaluating the potential causal association between WBC subtype counts and the risk of HCC. Our results suggest potential causal effects of lower sum basophil neutrophil counts on HCC occurrence in the European population. No evidence was found for causal effects of neutrophil counts, lymphocyte counts, monocyte counts, eosinophil counts, basophil counts, sum eosinophil basophil counts and sum eosinophil neutrophils on the risk of HCC.

It has been reported that most of HCC cases arise on the background of chronic inflammation. Infiltrating inflammatory cells is associated with tumour initiation, progression and clinical treatment response. Neutrophils are the most common leukocytes in circulation [21]. The increase in neutrophil counts changes the formation of the tumour microenvironment and inflammatory microenvironment, promoting HCC growth and metastasis, while neutrophils are reported to have antitumour potential [22]. Neutrophil-derived reactive oxygen species (ROS), such as nitric oxide and hydrogen peroxide, are cytotoxic towards cancer cells [23, 24]. Basophils play an important role in regulating innate immune response to infection and tissue repair. Gastric cancer-infiltrating basophils were identified as an independent adverse prognosticator [25]. However, a low percentage of basophils was associated with an increased number of pulmonary metastases in a breast cancer mouse model, which suggested that basophils had a protective role in breast cancer [26]. In ovarian cancer, higher basophil counts were positively correlated with improved prognosis [27]. Therefore, the role of basophils in the immune responses of cancers remains controversial.

Our univariable and multivariable analysis revealed that no causal association was observed between neutrophil counts, basophil counts and the occurrence of HCC based on the discovery and validation datasets. Our MR analysis suggested a seemingly increased risk of higher neutrophils in the multivariable models, while the sum (predominantly based on neutrophils) was associated with reduced HCC risk. The reason for this result may be that the SNPs associated with the sum (predominantly based on neutrophils) and those associated with neutrophils are largely different. For example, the top 5 SNPs with the largest effect value for neutrophils are rs56378716, rs12600856, rs56388170, rs11725704, and rs41313381. On the other hand, the top 5 SNPs with the largest effect value for sum were rs3812049, rs150425398, rs8028409, rs183034862, and rs17462448 (Sum basophil neutrophil counts) and rs34599082, rs114050631, rs445, rs4760, and rs1982094 (Sum neutrophil eosinophil counts).

Eosinophils play a prominent role in responses to allergic, inflammatory and immunoregulatory situations. A previous study reported that 0.5% of more than 2000 patients with malignant tumours of all histological types exhibited eosinophilia [28]. Eosinophils could promote the adverse effect of tumour metastasis [29]. Besides, eosinophilia may be a potential causal risk factor in squamous cell lung cancer progression in the East Asian population, while the association between eosinophil counts and lung cancer was not significant in the European population [30]. To date, there is no related literature to report the relationship between eosinophils and HCC. In the validation dataset, our univariable MR analysis showed that eosinophil counts had a significant association with HCC, suggesting a protective role of eosinophils for the risk of HCC. However, after adjustments for other traits, the association became nonsignificant. Several studies have reported that eosinophilic infiltration in HCC samples may be correlated with hepatocarcinogenesis and HCC progression [31–33]. The eosinophilic infiltration in HCC samples is different from the eosinophil counts in the blood used in our study.

Lymphocytes, which are usually recruited to the TME and engage in cell-mediated tumour responses, are associated with the prognosis of patients with various types of cancer [34, 35]. Peripheral lymphopenia can impair the host’s antitumour response and is conducive to tumour progression and dissemination [36]. Monocyte counts have been reported as prognostic markers for various cancers patients. Monocytes-derived tumour-associated macrophages are reported to be associated with cancer progression [37]. In HCC, low lymphocyte counts and high monocyte counts were significantly associated with reduced overall survival [9]. Our MR analysis based on two datasets found that neither lymphocyte counts nor monocyte counts were associated with the risk of HCC, which suggested that genetically predicted counts of most WBC subtypes might not be associated with HCC risk except the sum basophil neutrophil counts.

Our study attempted to reduce confounding bias through MR methods such as strict IV selection procedures, sensitivity tests and obtaining consistent findings in the two datasets. However, some limitations should not be ignored. First, because our datasets were both from European populations, the results should be further validated in other non-European ethnicities. Second, due to the limitation of the datasets, the association between genetically predicted NLR, LMR and HCC risk was not evaluated. Third, our MR analysis revealed no correlation between HCC risk and several exposures, such as the sum of basophils and eosinophils and the sum of neutrophils and eosinophils, which does not mean that these exposures have no effect on HCC development. These results may be related to the bias caused by the insufficient HCC sample size. Last, future studies are needed to clarify the mechanism of WBC subtypes in HCC occurrence.

## Conclusion

Our MR analysis suggests that genetically predicted sum basophil neutrophil counts are associated with the risk of HCC. This study provides a novel finding that European ancestry individuals who had lower genetic level counts of sum basophil neutrophils are likely at risk of HCC. Clinicians should raise awareness of total basophil neutrophil counts in clinical practice.

## Data Availability

The summary statistics of exposures were available on (https://gwas.mrcieu.ac.uk/); the summary statistics of outcome were available on (GWAS Catalog (ebi.ac.uk)).
